# Network pharmacological analysis of Xuefu Zhuyu decoction in the treatment of atherosclerosis

**DOI:** 10.3389/fphar.2022.1069704

**Published:** 2022-12-02

**Authors:** Jinxia Yuan, Fei Yan, Wei Li, Guoliang Yuan

**Affiliations:** ^1^ Department of Cardiology, Shuyang Hospital of Traditional Chinese Medicine, Yangzhou University, Shuyang, Jiangsu, China; ^2^ School of Medicine, Yangzhou University, Yangzhou, Jiangsu, China

**Keywords:** network pharmacology, molecular docking, xuefu zhuyu decoction, mechanism, atherosclerosis

## Abstract

**Objective:** Using a network pharmacological approach, this study will evaluate the effect of Xuefu Zhuyu Decoction in the treatment of atherosclerosis.

**Methods:** The data were imported into the STRING database to construct a protein-protein interaction network, and the network topology was analysed with the Bisogenet plug-in by Cytoscape 3.7.2. Using the R language Bioconductor platform, Gene Ontology (GO) enrichment analysis and Kyoto Encyclopedia of Genes and Genomes (KEGG) enrichment analysis for potential targets of Xuefu Zhuyu Decoction in the treatment of atherosclerosis were performed, and import the results were imported into Cytoscape 3.7.2. To map the results and create a KEGG network diagram, we used Cytoscape 3.7.2 for analysis.

**Results:** A total of 91 chemical components and 1320 disease targets were obtained, including 138 cross-targets. TNF, AKT1 and ALB were identified as important targets, and Gene Ontology functional analysis indicated that biological process was the primary cause of oxidative stress. The primary action of molecular function is binding. KEGG has explored and enriched 149 signalling pathways, including the AGE-RAGE signalling system and the TNF signalling network. According to a study involving molecular docking, quercetin and β-carotene have a strong binding affinity for AKT1 and ALB.

**Conclusion:** The potential of Xuefu Zhuyu Decoction to treat atherosclerosis through multiple components and targets provides a way to further study its mechanism.

## Introduction

Atherosclerosis (AS) is characterized by the accumulation of fat and fibrous particles, as well as calcification, in the principal arteries (Wang and Bennett, 2012). The creation of atherosclerotic plaques is initiated by the activation of endothelial cells, which leads to the constriction of blood vessels and the activation of inflammatory pathways, resulting in the formation of atherosclerotic plaques (Wu et al., 2017; Jebari-Benslaiman et al., 2022). These processes lead to cardiovascular difficulties and remain the leading cause of death globally. Endothelial dysfunction, the creation of fatty streaks, the formation of fibrous plaques, and the rupture of plaques are all under investigation as potential causes of AS. Clinical medical research has demonstrated that traditional Chinese medicine has preventative and therapeutic effects on AS, and the physiopathological and clinical characteristics of AS distinguish it from several other conditions (Saigusa et al., 2020; Biros et al., 2022). Traditional Chinese medicine is increasingly used in the clinical adjuvant treatment of atherosclerosis. Although their mechanism of action is still unclear, it has been confirmed that Chinese herbal medicine can play a variety of anti-atherosclerotic roles by regulating ROS in cells (Zhang et al., 2022). Also, when atherosclerosis is caused by diabetes, traditional Chinese medicine works well to control the NLRP3 inflammatory response (Yuan et al., 2022).

Using network pharmacology to investigate the mechanisms of TCM-disease interactions has proven useful. Through advanced target-disease interaction networks and bioinformatics research, network pharmacology, which is coherent with TCM as a whole, can identify potential mechanisms of prescription effects in disease therapy. This is because TCM components are intricate and multipurpose. Due to its virtual screening properties, however, it must be confirmed in cellular and/or animal experiments (Hao and Xiao, 2014; Pangkanon et al., 2021).

## Materials and methods

### Screening of target components of Xuefu Zhuyu decoction

Identification of active chemical constituents of Chinese herbal medicines using TCM systems pharmacology platforms TCMSP and TCMID (Tao Ren, Hong Hua, Angelica sinensis, Sheng Di Huang, Niu Keng each, Chuan Xiong, Radix Platycodon, Citrus aurantium, *Glycyrrhiza* each, and Chai Hu). The active components of Xuefu Zhuyu Decoction were identified by screening in TCMSP, with an oral bioavailability (OB) of 30% and drug-likeness (DL) of 0.18. The likely protein targets were extracted from the Swiss target prediction database with conditional probability better than 0.1, and the screened protein targets were assigned standardized gene names in the UniProt database.

### Screening for key atherosclerotic disease-related targets

By searching the Gene-Cards and OMIM databases using the term “atherosclerosis”, atherosclerosis target genes were identified. Based on the score value, the number of targets was limited. Targets with scores greater than the median are often classified as potential targets for disease, and genes with scores greater than 5 in GeneCards were integrated with the OMIM database and deweighted as atherosclerosis-related targets. The stronger the correlation between goals and illness, the higher the score.

### Target acquisition and venn diagramming

Using a Venn diagram, determining the intersection of the targets of Xuefu Zhuyu Decoction and the target points for atherosclerosis is the effective target point for Xuefu Zhuyu Decoction in the treatment of atherosclerosis.

### Analysing the active ingredient-target network

The active ingredients and effective target genes were imported into Cytoscape 3.7.2 software for network construction, visualization, and a drug-active ingredient-target network diagram was obtained.

### Construction of the protein network

The possible targets of the antiatherosclerotic effect of Xuefu Zhuyu decoction were entered into the GeneMANIA database (http://genemania.org), and their interactions were discovered. The indirect objectives were then acquired. The indirect targets have been added to the database of acting targets, which has been imported into the STRING database. To obtain the desired interaction network, *Homo sapiens*, who received a default score of 0.4, was utilized. Cytoscape 3.7.2 was used to analyze the network topology once the TSV format was imported and saved. After calculating the degree, betweenness centrality (BC), topological coefficient (TC), and closeness centrality (CC) of each node, the top 3 targets were selected as key target proteins.

### Analysis of target function enrichment and route expansion

Using R software (https://www.r-project.org) and its backend database org, gene IDs (entrezIDs) of possible targets were determined. The Hs.e.g.db database was utilized to perform GO functional enrichment analysis on these possible targets, followed by the Bioconductor tools DOSE, cluster Profiler, and path-view. The GO function enrichment analysis of these potential targets included three components: DOSE, cluster Profiler, and the path-view instrument (all from Bioconductor). We set *p* value Cutoff = 0.05 and q value Cutoff = 0.05 as appropriate for biological process (BP), cellular component (CC), and molecular function (MF). GO enrichment analysis revealed three fundamental categories: BP, MF and CC. The top ten items for enrichment are displayed as bar and bubble charts, with each category sorted in order of significance.

### Molecular docking of Xuefu Zhuyu decoction’s main active ingredient target

The targets of Xuefu Zhuyu Decoction acting on atherosclerosis were retrieved from the PDB database and saved in pdb format. After topological analysis, the compounds with the top 2 ligands in degree value were saved in mol2 format. The potential targets of Xuefu Zhuyu Decoction in atherosclerosis were molecularly docked with the main compounds of Xuefu Zhuyu Decoction using AutoDockTools-1.5.6.

## Results

### Obtaining the active components and objectives of Xuefu Zhuyu decoction

Xuefu Zhuyu Decoction includes 91 active constituents, as determined by screening criteria of OB 30% and DL 0.18, high GI absorption for pharmacokinetics, and over two “YESs” in druglikeness in TCMSP and TCMID. A total of 240 targets of Chinese medicine were retrieved by TCMSP and TCMID.

### Atherosclerosis-related target acquisition

Following the discovery of disease genes using GeneCards and Omim, 1320 atherosclerosis-related targets were identified, and targets with scores above the median were empirically classified as potential disease targets. Then, these targets were added to relevant targets found through searches of the OMIM database, and duplicate values were eliminated.

### Venn diagrams

**FIGURE 1:**
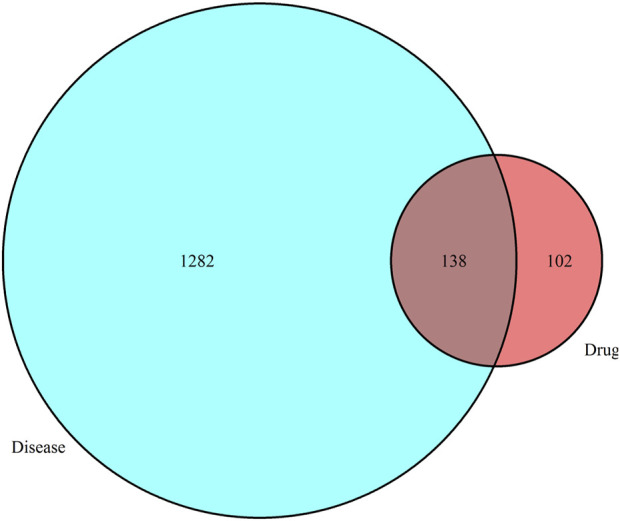
Venn diagram illustrating the overlap between Xuefu Zhuyu Decoction and atherosclerosis targets.

Using the Venn tool in TBtools, Figure 1 displays the intersection of the Xuefu Zhuyu Decoction and atherosclerosis targets.

### Network diagram of active targets for Xuefu Zhuyu decoction

**FIGURE 2:**
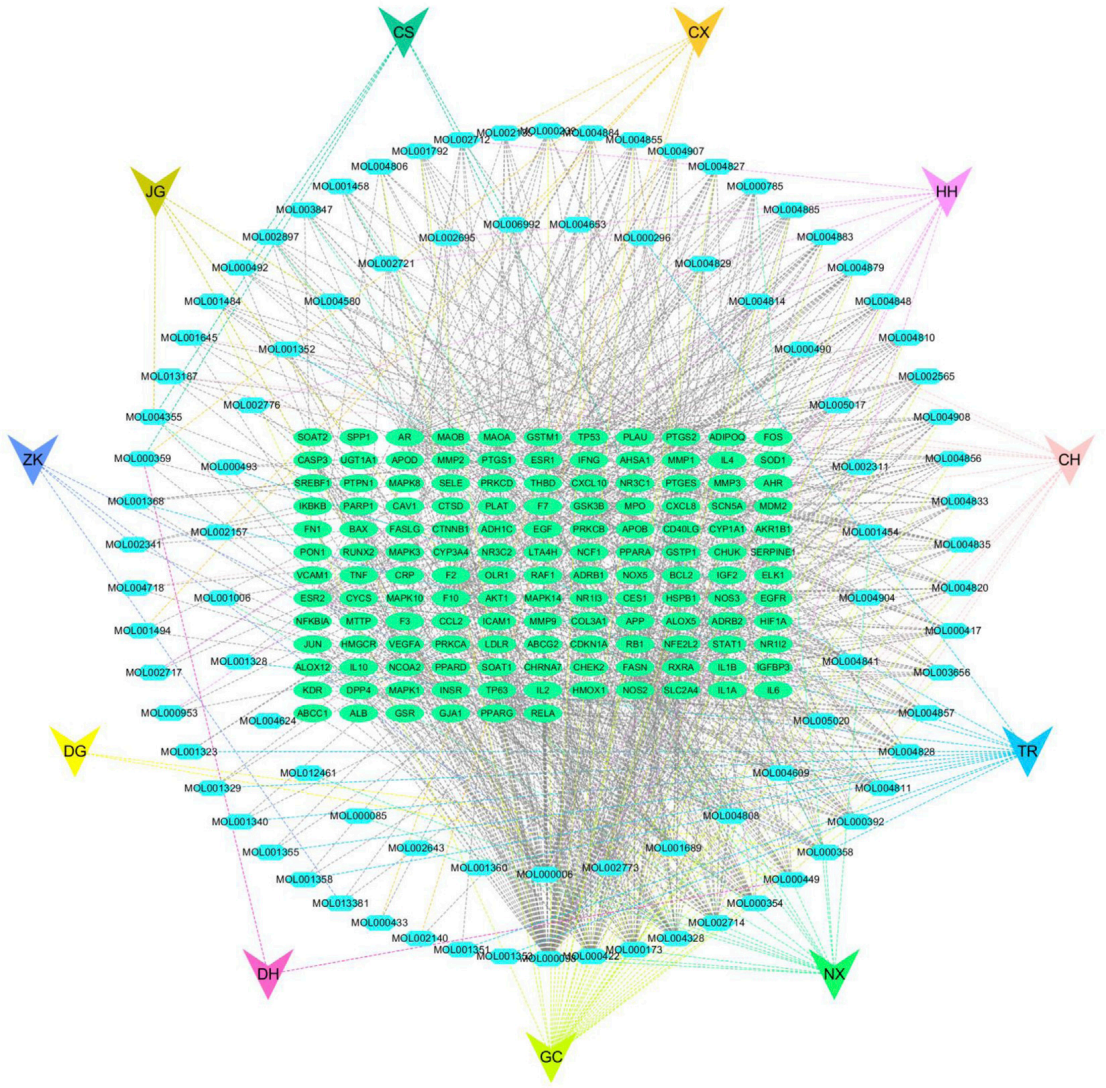
Active ingredient-active target network diagram of Xuefu Zhuyu Decoction.

Cytoscape 3.7.2 Active ingredients and an efficient target network of Xuefu Zhuyu Decoction. Software was used to calculate the topological parameters of the Xuefu Zhuyu Decoction network for the treatment of atherosclerosis to analyze the importance of activity components and action goals. Based on the findings, quercetin, β-carotene, and kaempferol may be the primary active components in Xuefu Zhuyu Decoction that help treat atherosclerosis by acting on multiple targets Figure 2.

### Construction of the protein network

**FIGURE 3:**
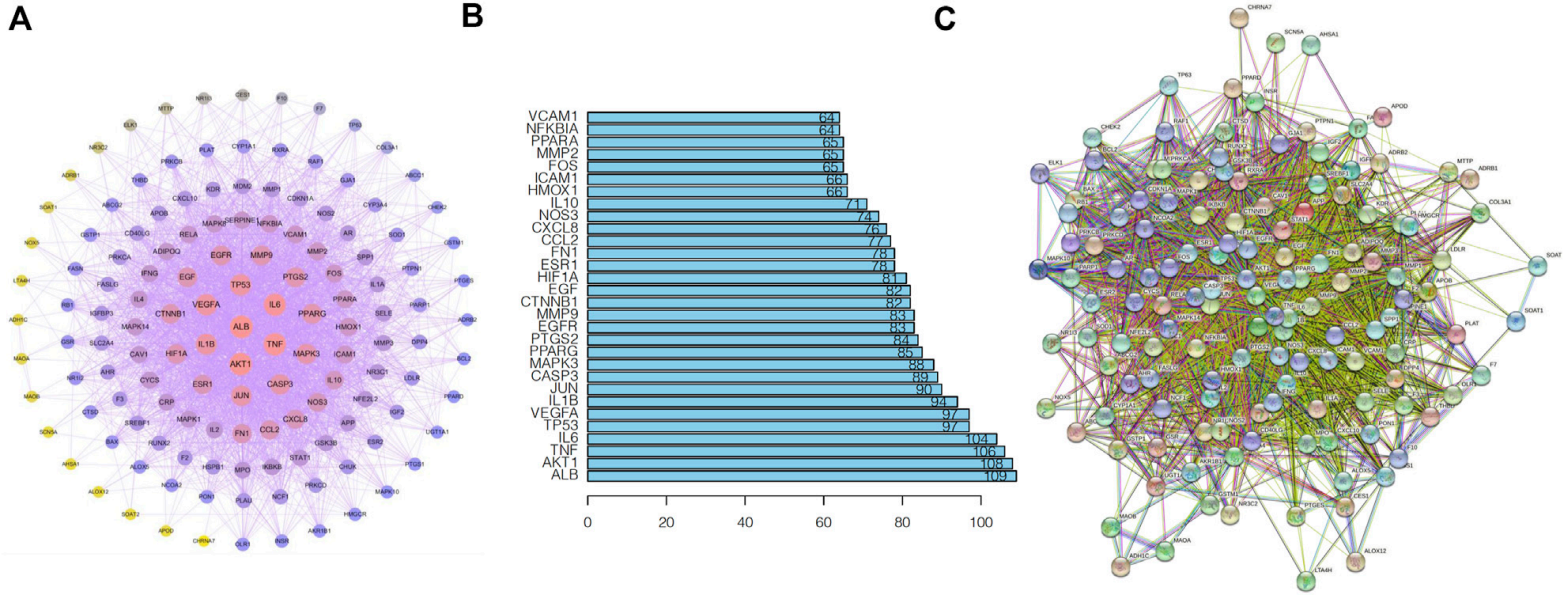
A schematic of the intersection target PPI network is shown. **(A)** PPI network map; **(B)** Core Gene Map; and **(C)** PPI Network Map.

We uploaded the intersection targets of Xuefu Zhuyu Decoction and atherosclerosis to the STRING database using the Venn tool, with the confidence level set to 0.90, and then built the PPI network map of targets. Cytoscape 3.7.2 was utilized to create the protein network relationship map. To decide network placement, the degree value size is used. The degree value is proportional to the size of the node. Haematopoeia deemed AKT1, ALB, IL6, TNF, TP53, and other targets important targets for the treatment of atherosclerosis (Figure 3).

### Results of an investigation of target pathway and function enrichment

**FIGURE 4:**
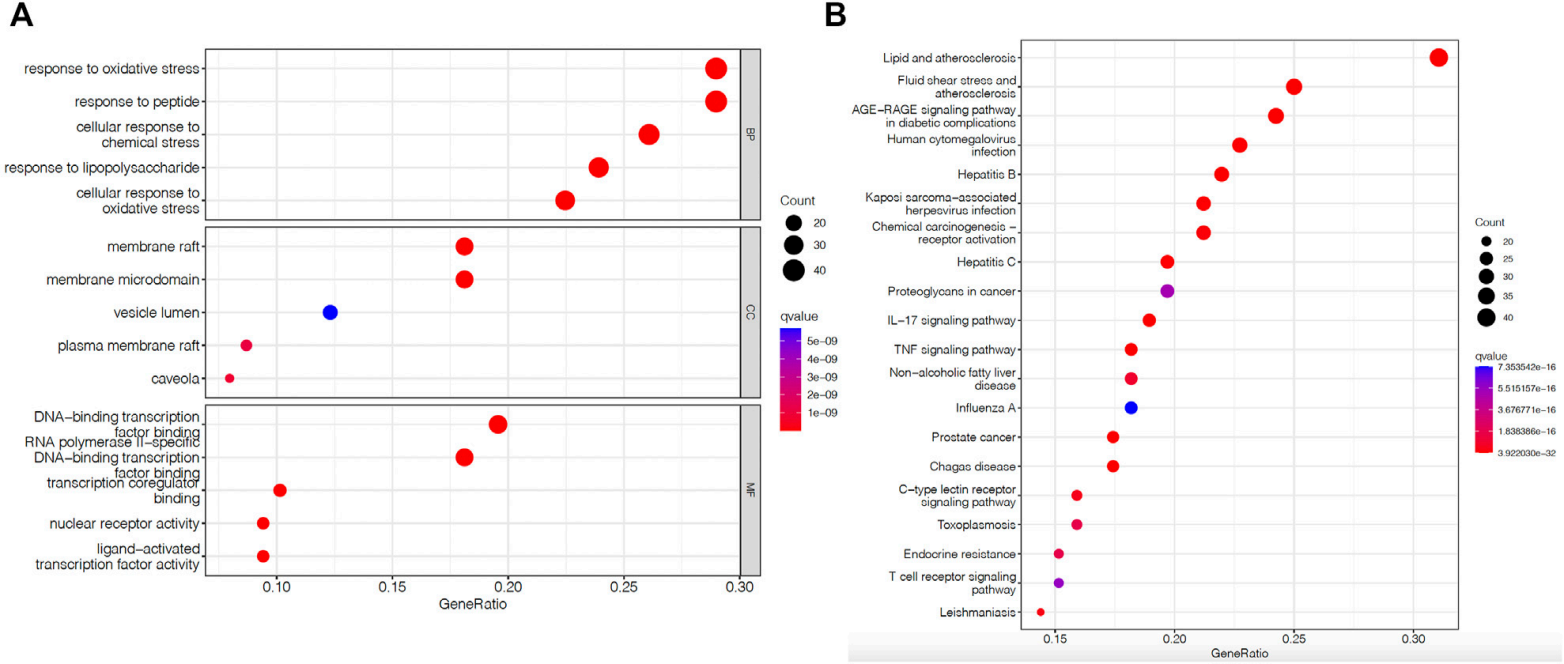
Enrichment analysis of Xuefu Zhuyu decoction in the treatment of atherosclerosis. **(A)** GO enrichment analysis; **(B)** KEGG enrichment analysis circle diagram.

GO annotation analysis of valid targets using R. The top ten results in BP, CC, and MF were selected, and oxidative stress was determined to be the major function of these target BPs. The primary functions of MF included binding activities. CC was predominantly active within the vesicle lumen, membrane raft, and microfilm areas. Figure 4A illustrates the results.

The top 20 KEGG-enriched signaling pathways visualized for analysis included the AGE-RAGE signaling route, the TNF signaling pathway, the IL-17 pathway, and others. Figure 4B demonstrates the outcomes.

### Molecular docking results of the active ingredients of Xuefu Zhuyu decoction

**FIGURE 5:**
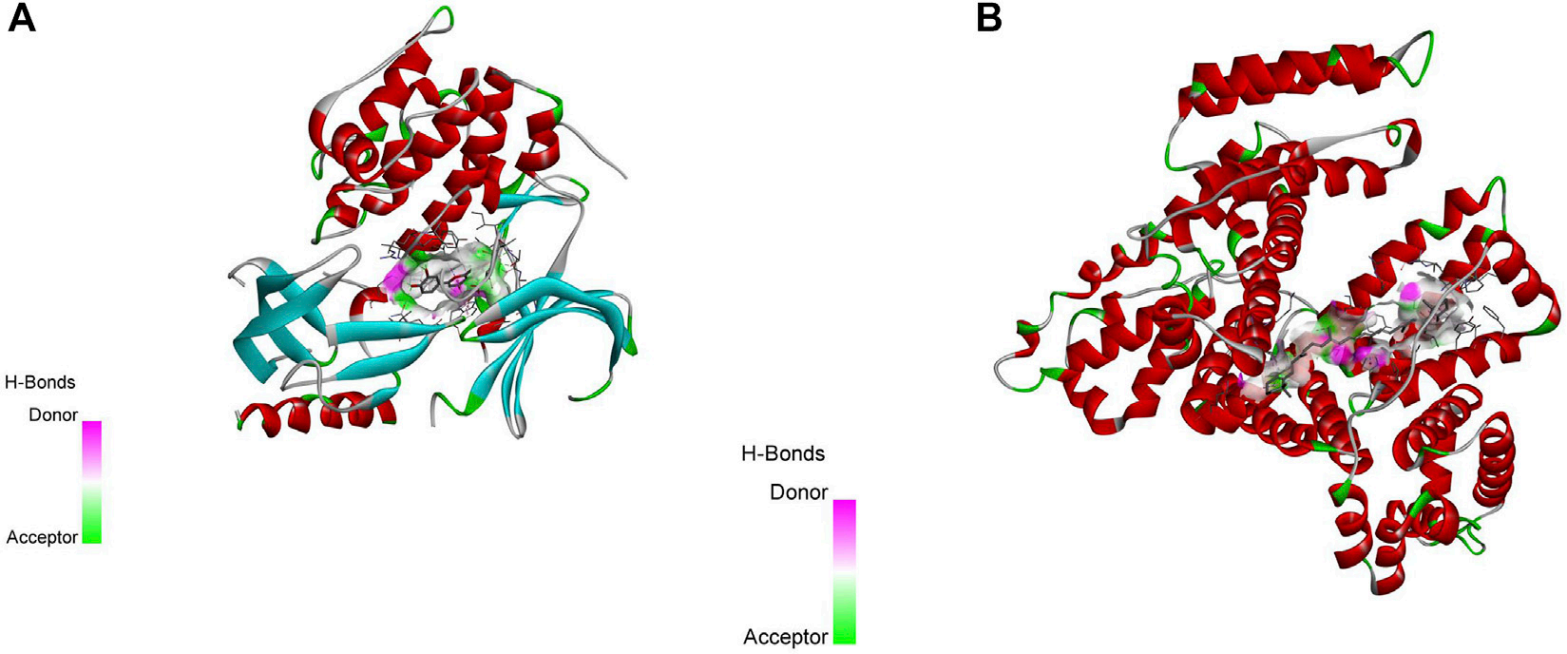
Diagram of Molecular docking model. **(A)** The docking diagram of quercetin and AKT1. **(B)** The docking diagram of β-carotene and ALB.

The key components of Xuefu Zhuyu Decoction identified by topological analysis were molecularly docked with the probable targets of Xuefu Zhuyu Decoction in atherosclerosis using AutoDockTools-1.5.6. The likelihood of action increased as the ligand‒receptor binding configuration became more stable, and the degree value was used to identify the most crucially important targets. A binding energy of -4.25 kcal/mol or less indicates some binding activities between ligand small molecules and receptor proteins, 5.0 kcal/mol or greater indicates good binding activity between the two, and -7.0 kcal/mol or greater indicates robust conjugation activity between ligand and receptor. According to the docking results in Figure 5, the binding energies of quercetin and β-carotene with the AKT1 and ALB core targets were -9.9 and -10 kcal/mol, respectively, showing strong binding activity between the drug and target sections.

## Discussion

Recent clinical research indicates that Chinese herbal medicine has special benefits for the development of anti-AS drugs and for enhancing the quality of patient survival. Several studies have already demonstrated the anti-inflammatory and antioxidant properties of herbal therapy in the aetiology of AS-7. Despite the fact that long-term use of statins can cause undesired side effects such as drug dependence and liver damage, oral statins are commonly used in the prevention and treatment of atherosclerosis (Gotto, 2003). Xuefu Zhuyu Decoction can circulate Qi and alleviate pain, stimulate blood circulation and remove blood clots, and induce blood to self-settle by removing blood clots. However, due to the molecular complexity of medicine, it is challenging to develop a therapeutic mechanism utilising basic research approaches (Cao, 2022).

This study screened 91 active compounds using a combination of bioinformatics and network pharmacology, providing 240 active ingredient action targets. In addition, 1320 disease gene targets were intersected to produce a total of 138 common targets, among which active components such as quercetin, -carotene, and kaempferol were revealed. This study provides a molecular mechanism for the use of Xuefu Zhuyu Decoction in the treatment of atherosclerosis. The findings of this study provide a more detailed explanation of how Xuefu Zhuyu Decoction’s can treat atherosclerosis at the molecular level and can be applied to make medicines to flight atherosclerotic-related illnesses.

A topological analysis of the ingredient-target-pathway network revealed that quercetin and β-carotene are key active ingredients in the treatment of atherosclerosis and correlate with more targets. It has been reported that quercetin, which blocked the Galectin-3-NLRP3 signaling pathway, reduced the inflammatory response caused by atherosclerosis (Li et al., 2021). Moreover, the transformation of beta-carotene into vitamin A slows down the evolution of atherosclerosis in mice by lowering the amount of hepatic lipid production (Zhou et al., 2020). According to the PPI network, ALB and AKT1 are the primary targets for Xuefu Zhuyu Decoction therapy to cure atherosclerosis.

AKT1 can participate in numerous signaling pathways, such as the cell cycle, inflammatory response, PI3K/Akt, MAPK, and AGE-RAGE signaling (Fernandez-Hernando et al., 2007; Hashimoto et al., 2017; Chen et al., 2019). Numerous studies have demonstrated that the NF-B pathway can regulate TNF and other inflammatory cytokines, enhancing endothelial function and reducing foam cell production and rupture, vascular smooth muscle cell proliferation, and atherosclerotic plaque growth (Ridker et al., 2017; Yang et al., 2019; Pangkanon et al., 2021). Interferon, a key immune function regulator, is highly expressed in atherosclerotic lesions, and mouse studies indicate that T and B-cell deficiency reduces the atherosclerotic burden during the development of atherosclerotic lesions and that regulation of the IL-17 signaling pathway may play a role in the pathogenesis of several inflammatory and autoimmune diseases, including atherosclerosis (Chen et al., 2010; Fan et al., 2016; Lu, 2017).

According to the results of the GO analysis, the therapy of the atherosclerotic pathway by Xuefu Zhuyu Decoction predominantly includes lipid metabolism and binding. Xuefu Zhuyu Decoction may aid in the treatment of AS by regulating the AGE-RAGE signaling cascade, TNF, and MAPK, according to a KEGG analysis.

A network pharmacology approach was utilized to evaluate the therapeutic targets and mechanisms of action of core Chinese herbal medicines on common-type atherosclerosis. Xuefu Zhuyu Decoction has anti-inflammatory and antioxidant properties *via* quercetin, β-carotene, kaempferol, and other flavonoid active component monomers. Consequently, it provides evidence to support its pharmacological impact. However, Network pharmacology is based on databases and analytical software, this study was analyzed through two databases, so the analysis results have limitations. The additional experimental validation is necessary to substantiate this assertion. In addition, the concentration used is not recorded in the database, but for the drug to work, it needs to be used at a certain concentration.

## Data Availability

The datasets presented in this study can be found in online repositories. The names of the repository/repositories and accession number(s) can be found in the article/Supplementary Material.

## References

[B1] Biros E. Reznik J. E. Moran C. S. (2022). Role of inflammatory cytokines in Genesis and treatment of atherosclerosis. Trends cardiovasc. Med. 32 (3), 138–142. 10.1016/j.tcm.2021.02.001 33571665

[B2] Cao R. (2022). Clinical observation on the prevention of deep vein thrombosis after total hip arthroplasty with the application of hemifu huang yu tang. J. Pract. Chin. Med., 1281–1283.

[B3] Chen L. Zheng S. Y. Yang C. Q. Ma B. M. Jiang D. (2019). MiR-155-5p inhibits the proliferation and migration of VSMCs and HUVECs in atherosclerosis by targeting AKT1. Eur. Rev. Med. Pharmacol. Sci. 23 (5), 2223–2233. 10.26355/eurrev_201903_17270 30915770

[B4] Chen S. Crother T. R. Arditi M. (2010). Emerging role of IL-17 in atherosclerosis. J. Innate Immun. 2 (4), 325–333. 10.1159/000314626 20505315 PMC2895754

[B5] Fan Z. Yang J. Yang C. Yang J. Guo X. (2016). IL-17: A promising therapeutic target for atherosclerosis. Int. J. Cardiol. 202, 930–931. 10.1016/j.ijcard.2015.08.195 26547663

[B6] Fernandez-Hernando C. Ackah E. Yu J. Suarez Y. Murata T. Iwakiri Y. (2007). Loss of Akt1 leads to severe atherosclerosis and occlusive coronary artery disease. Cell. Metab. 6 (6), 446–457. 10.1016/j.cmet.2007.10.007 18054314 PMC3621848

[B7] Gotto A. M. (2003). Antioxidants, statins, and atherosclerosis. J. Am. Coll. Cardiol. 41 (7), 1205–1210. 10.1016/s0735-1097(03)00082-2 12679223

[B8] Hao D. C. Xiao P. G. (2014). Network pharmacology: A rosetta stone for traditional Chinese medicine. Drug Dev. Res. 75 (5), 299–312. 10.1002/ddr.21214 25160070

[B9] Hashimoto R. Kakigi R. Nakamura K. Itoh S. Daida H. Okada T. (2017). LPS enhances expression of CD204 through the MAPK/ERK pathway in murine bone marrow macrophages. Atherosclerosis 266, 167–175. 10.1016/j.atherosclerosis.2017.10.005 29032172

[B10] Jebari-Benslaiman S. Galicia-Garcia U. Larrea-Sebal A. Olaetxea J. R. Alloza I. Vandenbroeck K. (2022). Pathophysiology of atherosclerosis. Int. J. Mol. Sci. 23 (6), 3346. 10.3390/ijms23063346 35328769 PMC8954705

[B11] Li H. Xiao L. He H. Zeng H. Liu J. Jiang C. (2021). Quercetin attenuates atherosclerotic inflammation by inhibiting galectin-3-NLRP3 signaling pathway. Mol. Nutr. Food Res. 65 (15), e2000746. 10.1002/mnfr.202000746 33939881

[B12] Lu X. (2017). The impact of IL-17 in atherosclerosis. Curr. Med. Chem. 24 (21), 2345–2358. 10.2174/0929867324666170419150614 28425862

[B13] Pangkanon W. Yenbutra P. Kamanamool N. Tannirandorn A. Udompataikul M. (2021). A comparison of the efficacy of silicone gel containing onion extract and aloe vera to silicone gel sheets to prevent postoperative hypertrophic scars and keloids. J. Cosmet. Dermatol. 20 (4), 1146–1153. 10.1111/jocd.13933 33387398 PMC8048999

[B14] Ridker P. M. Everett B. M. Thuren T. MacFadyen J. G. Chang W. H. Ballantyne C. (2017). Antiinflammatory therapy with canakinumab for atherosclerotic disease. N. Engl. J. Med. 377 (12), 1119–1131. 10.1056/NEJMoa1707914 28845751

[B15] Saigusa R. Winkels H. Ley K. (2020). T cell subsets and functions in atherosclerosis. Nat. Rev. Cardiol. 17 (7), 387–401. 10.1038/s41569-020-0352-5 32203286 PMC7872210

[B16] Wang J. C. Bennett M. (2012). Aging and atherosclerosis: Mechanisms, functional consequences, and potential therapeutics for cellular senescence. Circ. Res. 111 (2), 245–259. 10.1161/CIRCRESAHA.111.261388 22773427

[B17] Wu M. Y. Li C. J. Hou M. F. Chu P. Y. (2017). New insights into the role of inflammation in the pathogenesis of atherosclerosis. Int. J. Mol. Sci. 18 (10), E2034. 10.3390/ijms18102034 PMC5666716 28937652

[B18] Yang S. Li J. Chen Y. Zhang S. Feng C. Hou Z. (2019). MicroRNA-216a promotes M1 macrophages polarization and atherosclerosis progression by activating telomerase via the Smad3/NF-κB pathway. Biochim. Biophys. Acta. Mol. Basis Dis. 1865 (7), 1772–1781. 10.1016/j.bbadis.2018.06.016 29940270

[B19] Yuan J. Y. Fu Y. Feng Z. H. Sang F. Shao M. Y. Li L. L. (2022). Potential mechanisms and effects of Chinese medicines in treatment of diabetic atherosclerosis by modulating NLRP3 inflammasome: A narrative review. Chin. J. Integr. Med. 28 (8), 753–761. 10.1007/s11655-022-3513-4 35507299

[B20] Zhang L. Huang J. Zhang D. Lei X. Ma Y. Cao Y. (2022). Targeting reactive oxygen species in atherosclerosis via Chinese herbal medicines. Oxid. Med. Cell. Longev. 2022, 1852330. 10.1155/2022/1852330 35047104 PMC8763505

[B21] Zhou F. Wu X. Pinos I. Abraham B. M. Barrett T. J. von Lintig J. (2020). β-Carotene conversion to vitamin A delays atherosclerosis progression by decreasing hepatic lipid secretion in mice. J. Lipid Res. 61 (11), 1491–1503. 10.1194/jlr.RA120001066 32963037 PMC7604725

